# Effective fucoxanthin production in the flagellate alga *Poterioochromonas malhamensis* by coupling heterotrophic high-cell-density fermentation with illumination

**DOI:** 10.3389/fbioe.2022.1074850

**Published:** 2022-12-02

**Authors:** Hu Jin, Yufen Guo, Yanhua Li, Baofeng Chen, Haiyan Ma, Hongxia Wang, Lan Wang, Danni Yuan

**Affiliations:** ^1^ Institute of Hydrobiology, Chinese Academy of Sciences, Wuhan, China; ^2^ School of Life Science and Food Engineering, Huaiyin Institute of Technology, Huaian, China; ^3^ School of Environmental Ecology and Biological Engineering, Wuhan Institute of Technology, Wuhan, China

**Keywords:** fucoxanthin, heterotrophic, illumination-coupled fermentation, *Poterioochromonas malhamensis*, microalgae

## Abstract

The unicellular flagellate algae *Poterioochromonas malhamensis* is a potential fucoxanthin-rich resource for sustainable and cost-effective fucoxanthin production. Light and nutrients are critical regulators for the accumulation of fucoxanthin in *P. malhamensis*. In this study, the maximum fucoxanthin yield of 50.5 mg L^−1^ and productivity of 6.31 mg L^−1^ d^−1^ were achieved by coupling high-cell-density fermentation with illumination. It was found that the combined use of organic and inorganic nitrogen (N) nutrition could improve the fucoxanthin yield as single inorganic or organic N had limitation to enhance cell growth and fucoxanthin accumulation. White light was the optimal light quality for fucoxanthin accumulation. Under white light and a moderate light intensity of 150 μmol m^−2^ s^−1^, the highest biomass concentration and fucoxanthin content reached 32.9 g L^−1^ and 1.56 mg g^−1^ of dry cell weight (DCW), respectively. This is the first study on effective fucoxanthin production in *P. malhamensis* by integrating illumination with high-cell-density fermentation, which paved the way for further development of *P. malhamensis* as a potential source for commercial fucoxanthin production.

## Introduction

Fucoxanthin, derived mainly from brown macroalgae and marine microalgae, is one of the most plentiful carotenoid pigments, accounting for over 10% of the estimated total natural production of carotenoids (Wang et al., 2021a). Different from the plant accessory carotenoids of β-carotene and lutein, the unique molecule structure of fucoxanthin endows it exceptional light harvesting and photoprotection capabilities (Mikami & Hosokawa, 2013; Leong et al., 2022). Fucoxanthin possesses a broad therapeutic activities and it can be employed as a functional ingredient in cosmetics, nutraceuticals, pharmaceuticals, aquaculture, and poultry industries (Xia et al., 2013; Kanamoto et al., 2021). The market size of fucoxanthin was 99 million dollars in 2017 and is estimated to reach 120 million dollars by 2022 (Pajot et al., 2022).

Currently, the primary feedstock for commercial fucoxanthin production is brown macroalgae. However, industrial production of fucoxanthin from these natural resources is costly and unsustainable owing to their inherent shortcomings including low fucoxanthin content, seasonal growth, limited resource, and long cultivation period (Li et al., 2019; Zhang et al., 2022). Compared to brown macroalgae, microalgae are viewed as more promising commercial fucoxanthin producers because of their more abundant fucoxanthin content, higher growth rate, and shorter cultivation duration (Ishika et al., 2017; Wang et al., 2018; Xia et al., 2018; Mohamadnia et al., 2020; Wang et al., 2021a). However, despite the great potentials of microalgae for commercial fucoxanthin production, it is still in the infancy and has been rarely implemented worldwide (Wang et al., 2021a).

A cost-effective fucoxanthin production by microalgae relies on the achievement of both high fucoxanthin content and high biomass concentration. However, for most of the widely investigated fucoxanthin-rich microalgae including *Phaeodactylum tricornutum*, *Odontella aurita*, *Isochrysis sp.*, and *Mallomonas sp.*, large-scale fucoxanthin production by these microalgae in photobioreactors will be impeded by the difficulty to achieve high biomass concentration due to light limitation (Lu et al., 2018). Although microalgal biomass concentration and fucoxanthin productivity could be improved through mixotrophic cultivation, it is still challenged by limited species, contamination, and high production cost (Patel et al., 2021). Heterotrophic cultivation of microalgae eliminates the dependence on light and the microalgal cell density and productivity could be markedly improved (Chen & Jiang, 2017). For the heterotrophy microalga *Nitzschia laevi*, a superior fucoxanthin productivity of 16.5 mg L^−1^ d^−1^ was achieved by using a sequential heterotrophic and photoautotrophic culture strategy (Lu et al., 2018).

The unicellular flagellate algae *Poterioochromonas* spp. were another class of microalgae capable of producing fucoxanthin. It has been found that 75–78% of the total carotenoids was fucoxanthin when culturing *P. malhamensis* under continuous illumination (Withers et al., 1981). However, *P. malhamensis* has been rarely studied as a potential fucoxanthin-rich resource due to its poor growth under photoautotrophic cultivation. It was reported that photosynthesis in *P. malhamensis* was not sufficient to provide all the organic compounds required for multiplication, but it could grow rapidly by using organic carbon sources under dark heterotrophic cultivation (Andersson et al., 1989). We previously isolated a new *P. malhamensis* strain (*P. malhamensis* CMBB-1) and found it could accumulate large amount of β-1,3-glucan when using inorganic-N nutrition under dark heterotrophic cultivation. We further established its high-cell-density fermentation process and achieved the highest β-1,3-glucan yield of 13.9 g L^−1^ (Ma et al., 2021). Illumination is thought to be an effective strategy for inducing the accumulation of fucoxanthin in microalgae (Lu et al., 2018). It was found that *P. malhamensis* cells cultured in the dark were much less pigmented and usually almost colorless compared to brown cells in the light (Pringsheim, 1952), suggesting that light might be necessary for the accumulation of pigments in *P. malhamensis*. We speculated that *P. malhamensis* CMBB-1 might be a promising producer for commercial fucoxanthin production if effective fucoxanthin accumulation could be achieved by the combination of high-cell-density fermentation with illumination.

In the present work, the impact of illumination on *P. malhamensis* CMBB-1 growth, fucoxanthin production and cellular biochemical composition was investigated under different type of N sources during high-cell-density cultivation in fermentors. Compared to single organic and inorganic N source, the combined use of organic and inorganic N nutrition was found to be able to markedly improve fucoxanthin yield. Subsequently, the optimal culture condition for fucoxanthin accumulation was obtained after sequential optimization on light quality and light intensity. To the best of our knowledge, this is the first report on integrating high-cell-density fermentation with illumination for fucoxanthin production by *P. malhamensis*, which laid a foundation for the exploitation of *P. malhamensis* as candidate for commercial fucoxanthin production.

## Materials and methods

### Microalgal strain and culture conditions

The flagellate *P. malhamensis* CMBB-1 was isolated from the contaminated *Chlorella sorokiniana* GT-1 culture and was preserved in the China General Microbiological Culture Collection Center (No. 11620). For maintenance, 1 ml axenic *P. malhamensis* cell cultures were inoculated into 250 ml flasks containing 100 ml seed medium (per liter, 10.0 g glucose, 0.5 g KH_2_PO_4_, 1.0 g liver extract powder, 3.0 g yeast extract, and 0.5 g MgSO_4_·7H_2_O) and maintained in dark at 25°C. For seed cultivation, 20 ml of the maintained *P. malhamensis* cell cultures after 10 days’ static cultivation were inoculated to 500 ml flasks containing 200 ml seed medium and grown in dark at 28°C/180 rpm for 4 days, which was then used as inoculum for fermentation cultivation.

Fermentation cultivation of *P. malhamensis* CMBB-1 were performed in 1-L glass fermenters (MiniBio-1000 ml, Applicon, Netherlands) containing 0.7 L initial basal inorganic-N or organic-N medium (Ma et al., 2021). The basal inorganic-N medium contained, per liter, 10.0 g glucose, 0.5 g MgSO_4_·7H_2_O, 0.3 g KH_2_PO_4_, 1.0 g NH_4_Cl, 0.074 g CaCl_2_·2H_2_O, 4.40 mg Na_2_·EDTA, 0.97 mg H_3_BO_3_, 3.15 mg FeCl_3_·6H_2_O, 0.18 mg MnCl_2_·4H_2_O, 0.02 mg ZnSO_4_·7H_2_O, 0.012 mg Co(NO_3_)_2_·6H_2_O, 0.006 mg NaMoO_4_·2H_2_O, 0.375 mg vitamin B1 (VB1), and 0.125 mg vitamin B12 (VB12). For the basal organic-N medium with the same total N content of 0.26 g L^−1^, 1.0 g L^−1^ NH_4_Cl in the inorganic-N medium was replaced with 0.30 g L^−1^ liver extract and 0.94 g L^−1^ yeast extract. For the combined organic and inorganic N cultivation experiment, half inorganic-N (0.5 g L^−1^ NH_4_Cl) and organic-N (0.15 g L^−1^ liver extract and 0.47 g L^−1^ yeast extract) were used to keep the total N content constant. The inorganic-N feeding medium (per liter, 400.0 g glucose, 20 g MgSO_4_·7H_2_O, 12 g KH_2_PO_4_, 40 g NH_4_Cl, 2.96 g CaCl_2_·2H_2_O, 176 mg Na_2_·EDTA, 38.8 mg H_3_BO_3_, 126 mg FeCl_3_·6H_2_O, 7.2 mg MnCl_2_·4H_2_O, 0.8 mg ZnSO_4_·7H_2_O, 0.48 mg Co(NO_3_)_2_·6H_2_O, 0.24 mg NaMoO_4_·2H_2_O, 15 mg VB1, and 5 mg VB12) was the 40-fold concentrated basal inorganic-N medium. For the organic-N feeding medium, 40 g NH_4_Cl in the inorganic-N feeding medium was replaced with 12 g liver extract and 37.6 g yeast extract. Whereas for the mixed-N feeding medium, 40 g NH_4_Cl in the inorganic-N feeding medium was replaced with 20 g NH_4_Cl, 6 g liver extract and 16.8 g yeast extract.

The starting biomass concentration of each fermentation run was ∼0.3 g L^−1^ by inoculating ∼70 ml inoculum from the above seed cultivation. For the illumination-coupled fermentation cultivation, commercial LED lamp belts (https://item.jd.com/10034513212505.html) were winded around the glass fermentor (Supplementary Figure S1). LED lamp belts with different light quality were used for the light spectrum experiments and their emission light spectra were measured with a spectrometer (HR-450, HiPoint, Taiwan) (Supplementary Figure S2). Light intensity was adjusted by changing the number of cycles of the winded LED lamp belts. Here, light intensity in the fermentor was defined as the measured average value of the fermentor central axis. For the control experiment (dark heterotrophic cultivation), the glass fermentors were coated with aluminum clothes (Supplementary Figure S1).

For the illumination-coupled fermentation cultivation, continuous illumination was supplied during the whole fermentation period of 192 h. Except illumination, all the cultural conditions in fermentors were kept the same for both dark and illumination-coupled fementation cultivation. The culture pH was constantly controlled at 6.0 using 1 M HCl or 3 M NaOH. The culture temperature was controlled at 28°C and a close-cycle cooling chiller (LX-300, Beijing, China) was connected to the fermentor system to help cooling and maintain the fermentation temperature constant. Airflow rate and initial stirring speed were set at 0.5 L min^−1^ and 200 rpm, respectively. Dissolved oxygen (DO) was maintained above 20% by cascade controls of stirring speed. Glucose feeding was started when the measured glucose reduced below 5 g L^−1^. Glucose concentrations were maintained in 2–5 g L^−1^ by adjusting feeding rate as described previously (Jin et al., 2020).

### Determination of biomass concentration, cell number and glucose concentration

Biomass concentration was determined with GF/C filter paper as described in our previous report (Jin et al., 2021). Briefly, 1 ml of the culture was collected to centrifuge at 4,000 rpm for 3 min. Then the pellets were washed twice with deionized water and filtrated with the dry pre-weighed GF/C filter paper (Whatman, Maidstone, United Kingdom). The filter papers were then dried in a vacuum drying oven at 105°C to a constant weight. Finally, the filter papers were subsequently cooled down to room temperature and the weight was measured. The biomass concentration was expressed as dry cell weight (DCW) per liter. Cell number was counted with a hemocytometer (Improved Neubauer, United States) at 400× magnification as described previously (Ma et al., 2021). A blood glucose monitor (Safe AQ Smart, Sinocare, China) was used to measure the glucose concentration.

### Quantification of β-1,3-glucan, protein and lipid contents

The *P. malhamensis* cells were first treated with endo-1,3-beta-D-glucosidase and exo-1,3-β-D Glucanase/β-Glucosidase according the method reported previously (Becker et al., 2017). Then, β-1,3-glucan content was quantified based on the measured glucose concentration in the enzymatic hydrolysate with the method described previously (Lin et al., 2020). Protein content was measured with the Kjeldahl analyzer as described previously (Xu et al., 2021). Total lipids of *P. malhamensis* samples were extracted and measured as reported previously (Laurens et al., 2012). Briefly, 200 mg lyophilized *P. malhamensis* sample was ground with liquid nitrogen and extracted with 10 ml methanol and chloroform mixture (1:2, v/v) at 30°C for 60 min. After the remove of crude protein by adding 2.5 ml 0.7% (w/v) KCl and centrifuging at 2,000 g for 5 min, the lower chloroform phase containing crude lipids was collected and weighed after evaporated with nitrogen.

### Analyses of fatty acids profiles and content

For the determination of fatty acids, the lipids were first derived at 85°C for 60 min and the resulting fatty acid methyl esters (FAMEs) were analyzed using a gas chromatograph-mass spectrometry with the same instrument type and operation procedure as described in our previous study (Wu et al., 2019). Briefly, the lipids were dissolved in 10 μl of chloroform: methanol (1:1, v: v) and then mixed with 200 μL of chloroform: methanol (2:1, v: v), 300 μl of 5% HCl: methanol and 25 μl of C13:0 (200 μg ml^−1^). The mixtures were heated at 85°C for 1 h. After cooling down to room temperature, 1 ml hexane was added followed by a brief centrifugation. Two-hundred microliters of hexane top layer was transferred into a GC vial with insert, and then mixed with 5 μl Pentadecane standard (to achieve a final concentration of 200 μg ml^−1^) for FAME composition and content analysis, which was performed by using an Agilent 7890B gas chromatograph coupled with 5977A mass spectrometry (GC-MS) and capillary column HP-88 (60 m × 0.25 mm × 0.2 μm, Agilent). The spectrometry was set to scan in the range of m/z 50–500 at 70 eV with electron impact (EI) mode of ionization. The 37-Component FAMEs Mix standard (CRM 47885) was purchase from Merck.

### Extraction and analysis of pigments

The algal cells were harvested by centrifugation at 3,000 g for 5 min and washed twice in PBS buffer solution. The cell pellets were freeze-dried to completely remove water. Pigments were extracted by using methanol/dichloromethane (3:1, v/v) till the residues became colorless. The extracts were centrifuged at 12,000 × g for 5 min to remove the debris, and the supernatants were dried, recovered in 0.5 ml of extraction solution, and filtrated through 0.22 μm membrane filters (Pall Life Science, United States) before High-Performance Liquid Chromatography (HPLC) analysis. All the pigments were analyzed using a reversed-phase high-performance liquid chromatography (RP- HPLC) as previously described (Yuan et al., 2002). Briefly, a Waters e2695 liquid chromatography coupled with a 2998 photodiode array detector was used. All pigments were separated on a Waters Symmetry C18 column (5 μm; 150 × 4.6 mm) at room temperature. The mobile phase constituted of two mobile phases (A, B), eluent A (dichloromethane: methanol: acetonitrile: water, 5:85:5.5:4.5, v/v) and eluent B (dichloromethane: methanol: acetonitrile: water, 25:28:42.5:4.5, v/v). Separation of pigments was performed by the following gradient procedure: 0% of B for 8 min; a linear gradient from zero to 100% of B within 6 min; 100% of B for 40 min, at a flow rate of 1.0 ml/min. The absorption spectra of pigments were displayed between 250 and 700 nm. Peaks were measured at a wavelength of 480 nm for carotenoids detection. All pigments were identified by pigments standards and the UV absorption spectral properties of pigments. All pigments standards, including Chlorophyll a, β-carotene, lutein, and fucoxanthin were purchased from SigmaAldrich (St. Louis, MO, United States). Data are shown as means ± SD for three biological replicates.

### Statistic analysis

The results were analyzed based on the data from three biological independent replicates. Experimental results were expressed as mean value ±standard deviation (SD). The data were analyzed by using one-way ANOVA with SPSS (version 19.0). Statistically significant difference was considered at *p* < 0.05.

## Results and discussion

### Effects of light and type of N nutrition on *P. malhamensis* growth, β-1,3-glucan production and fucoxanthin accumulation

**FIGURE 1:**
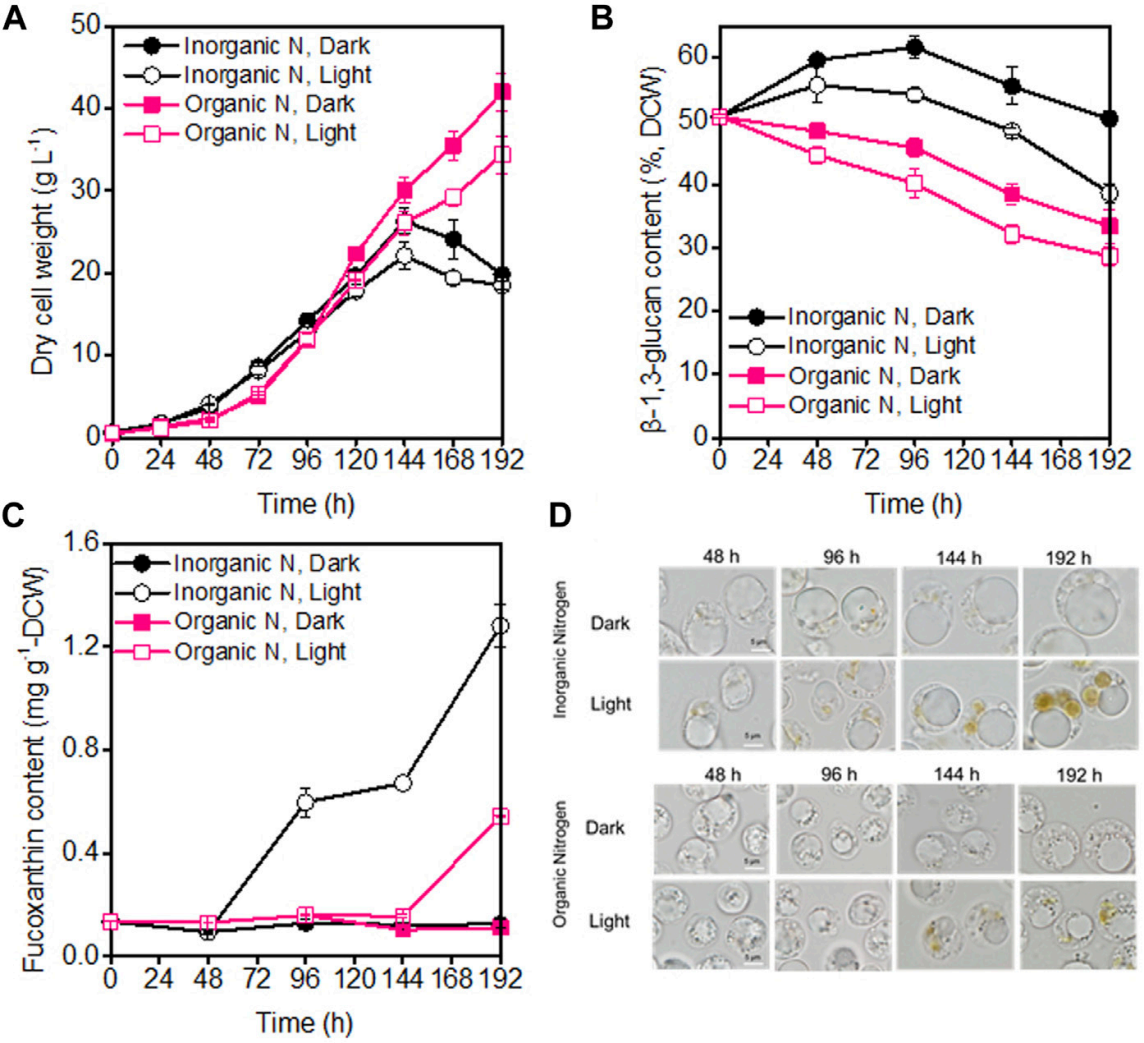
Comparison of biomass concentration **(A)**, β-1,3-glucan content **(B)**, fucoxanthin content **(C)**, and microscopic morphology of *P. malhamensis* cells **(D)** under dark and illumination-coupled fermentation cultivation (white light and light intensity of 50 μmol m^−2^ s^−1^) with single organic and inorganic N source. Data for **(A)** to **(C)** represent means ± SD (*n* = 3 biological replicates).

Under both dark and illumination-coupled fermentation, culturing *P. malhamensis* with inorganic-N exhibited faster growth rate for the first 96 h compared to organic-N source (Figure 1A). After 96 h, more rapid and sustained cell growth were observed when cultured with organic-N source. The highest biomass concentration under dark organic-N cultivation reached 42 g L^−1^, which were 60% higher compared to dark inorganic-N cultivation (Figure 1A). Similarly, for the cultivation of marine chlorophyte *Tetraselmis* sp., higher biomass concentration was achieved under organic nitrogen sources of yeast extract and glycine, compared to inorganic nitrate-N or ammonium-N (Kim et al., 2016). Light was found to have negative effect on cell growth for both N sources. As shown in Figure 1A, after 96 h, the biomass concentrations under illumination were obviously lower than those under dark cultivation (*p* < 0.05). Similar results have also been observed for many other microalgae (Chojnacka & Noworyta, 2004; Heredia-Arroyo et al., 2010; Lu et al., 2018). The reduced *P. malhamensis* growth under illumination might be caused by light-induced cell damage under the glucose-replete condition (Lu et al., 2018). Different from the continuous increase of biomass concentration under organic-N cultivation, *P. malhamensis* grown under inorganic-N nutrition groups showed a decreased trend after 144 h (Figure 1A), which might be caused by the following two main reasons. First, during the whole fermentation, the stirring speed increased gradually to maintain the DO level with the increased biomass concentration and oxygen requirement. After 120 h, the stirring speed has reached as high as 400 rpm, thus the high stirring speed during the late growth phase might increase the shearing damage on the naked *P. malhamensis* cells and cause the decline of cell growth. Second, it has been observed that the *P. malhamensis* cells grown under inorganic-N had much larger cell size/volume over those under organic-N cultivation (Figure 1D), which making them more easily to be broken when subjected to the same high shearing environment during the late growth phase.

Besides the influence on cell growth, light and the type of N sources also affected the production of β-1,3-glucan. The maximum β-1,3-glucan accumulation was observed under dark inorganic-N cultivation, where its content during the whole fermentation could maintain above 50% of DCW and the maximum content reached as high as 61.6% of DCW (Figure 1B). The obtained higher β-1,3-glucan contents under inorganic-N could also be well reflected from the microscopic observation of *P. malhamensis* cells. Compared to organic-N nutrition, *P. malhamensis* cells cultured under inorganic-N had larger volume of vacuole for β-1,3-glucan storage (Figure 1D). It appears that the introduction of illumination could accelerate the degradation of β-1,3-glucan. Faster decrease in β-1,3-glucan content was observed under illuminated cultivation, and the β-1,3-glucan content under illuminated organic-N cultivation reduced from initial 50.7%–28.6% at the end of fermentation (Figure 1B). It has also been found that light could induce the degradation of storage carbohydrate, and marked decrease in starch was observed when *Chlorella* cells were shifted from heterotrophic to photoautotrophic cultivation (Fan et al., 2012; Fan et al., 2015). Besides starch, another storage polysaccharide paramylon in *Euglena gracilis* was reported to be rapidly degraded in the presence of light (Hasan et al., 2019).

During high-cell-density cultivation, the introduction of illumination could induce the synthesis of pigments in *P. malhamensis* cells. HPLC analysis indicated that fucoxanthin was the main pigment (Supplementary Figure S3), which was consistent with the previous report (Withers et al., 1981). Besides light, the type of N nutrition also affected the synthesis of fucoxanthin. Under the same medium N concentration, inorganic-N was more suitable for fucoxanthin accumulation, which could be well reflected from the comparisons of broth color (Supplementary Figure S4) and microscopic observation of cells during the whole fermentation. It was observed that under illuminated inorganic-N cultivation, brown pigmentation developed gradually with culture time and more brown plastids were observed in *P. malhamensis* cells (Figure 1D). Under inorganic-N, the color of culture broth under illumination cultivation has become dark brown at 120 h, which was much darker than that under dark cultivation. Whereas under organic-N, although the color of culture broth changed gradually with culture time, the color difference in broth between dark and illumination cultivation for the same time was not obvious during the whole fermentation (Supplementary Figure S4). The highest fucoxanthin content under illuminated inorganic-N cultivation reached 1.28 mg g^−1^ DCW, which was 137% higher than the highest content (0.54 mg g^−1^ DCW) obtained under illuminated organic-N cultivation (Figure 1C). Among all the tested types of N nutrition (urea, sodium nitrate, ammonium chloride, and potassium nitrate), sodium nitrate was found to be the optimal N source for fucoxanthin production, and the maximum fucoxanthin titer of 17.8 mg L^−1^ was achieved from *Pavova sp*. OPMS 30543 (Kanamoto et al., 2021). Therefore, besides ammonium chloride, the effects of other defined N sources such as urea and sodium nitrate on *P. malhamensis* growth and fucoxanthin production would be further evaluated in future study.

**FIGURE 2:**
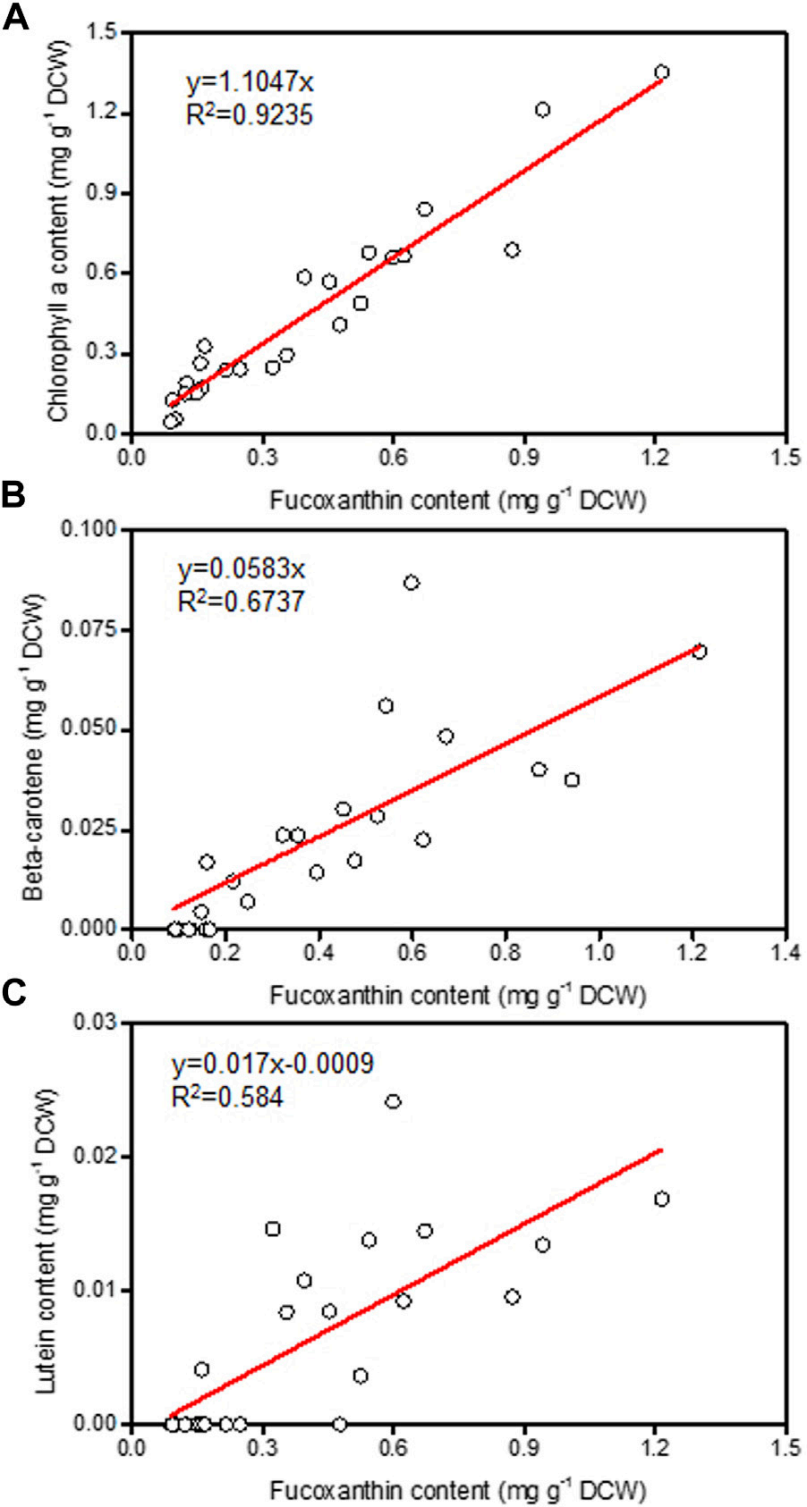
Relationship of fucoxanthin content with chlorophyll a **(A)**, beta-carotene **(B)**, and lutein **(C)** from different *P. malhamensis* pigment samples.

Nitrogen is not only the main nutrient for the growth of microalgae cells, it also plays a crucial role in the regulation of fucoxanthin synthesis (Guo et al., 2016). It was found that the supply of nitrogen could promote the accumulation of chlorophyll and then increase the biosynthesis of fucoxanthin (Leong et al., 2022). By analyzing the relationship between fucoxanthin content and the levels of other pigments (chlorophyll a, beta-carotene, and lutein) in different samples, the fucoxanthin content had strong positively linear correlation with chlorophyll a content (Figure 2), indicating that the *P. malhamensis* cells under illuminated organic N cultivation had less chlorophyll a content than those cultured under inorganic N nutrition. Compared to defined inorganic-N nutrition, complex organic-N source contains various amino acids, vitamins, nucleotides, and other active biomacromolecules, which can be used directly by *P. malhamensis* (Ma et al., 2021). Thus, under the same total N concentration with inorganic-N source, more N in the organic-N source might be used for the fast growth and less organic-N was decomposed for chlorophyll synthesis, thereby lower fucoxanthin accumulation was observed under organic-N cultivation.

### Improved fucoxanthin production by the combined use of inorganic and organic N sources under illumination-coupled fermentation

**FIGURE 3:**
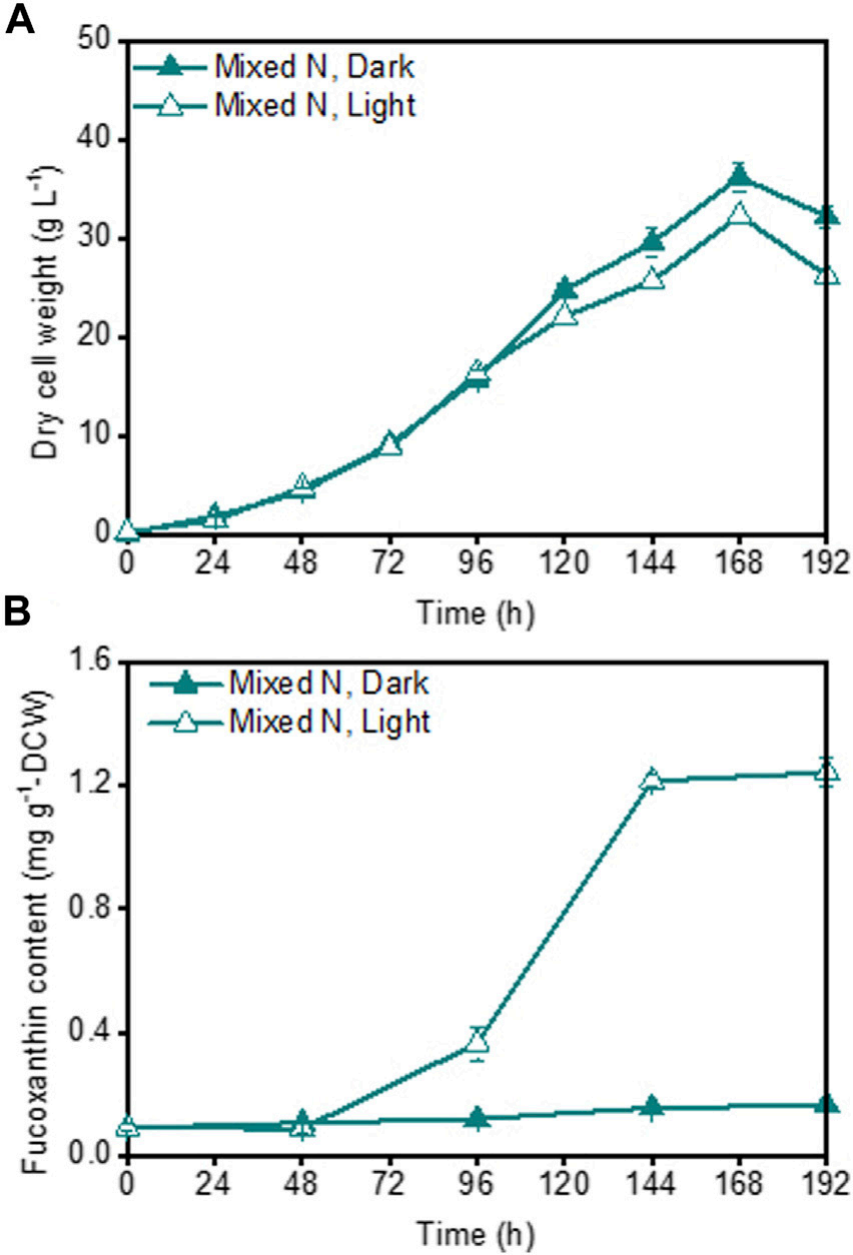
Effects of mixed organic and inorganic N source on *P. malhamensis* growth **(A)** and fucoxanthin content **(B)** under dark and illumination-coupled fermentation cultivation (white light and light intensity of 50 μmol m^−2^ s^−1^). Data for **(A)** and **(B)** represent means ± SD (n = 3 biological replicates).

Although a high fucoxanthin content could be achieved under illuminated inorganic-N fermentation cultivation (Figure 1C), the poor growth of *P. malhamensis* under inorganic-N nutrition hindered the further improvement of fucoxanthin production. In view of the beneficial effect of organic-N on *P. malhamensis* growth and inorganic-N on fucoxanthin accumulation (Figure 1), we speculated that their combination might improve the fucoxanthin production. Therefore, the impact of mixed N nutrition (combined organic and inorganic N sources with equal amount of N weight) on *P. malhamensis* growth and fucoxanthin production was evaluated. The results indicated that compared to inorganic-N alone, mixed-N nutrition could significantly improve *P. malhamensis* growth, and the maximum biomass concentration under dark mixed-N cultivation reached 36.2 g L^−1^, which increased 37.6% compared to that with dark inorganic-N cultivation (Figures 1A, 3A). Moreover, the maximum biomass concentration (32.3 g L^−1^) under illuminated mixed-N cultivation was 46.1% higher than that under illuminated inorganic-N cultivation and was close to the obtained highest biomass concentration (34.4 g L^−1^) under illuminated organic-N cultivation (Figures 1A, 3A). Under illuminated mixed-N cultivation, the highest fucoxanthin content reached 1.24 mg g^−1^ DCW, which was close to the highest fucoxanthin content (1.28 mg g^−1^ DCW) achieved from inorganic-N alone cultivation (Figures 1C, 3B). Consequently, the highest fucoxanthin yield and productivity under illuminated mixed-N cultivation reached 43.80 mg L^−1^ and 5.48 mg L^−1^ d^−1^, respectively, which increased 84.8% and 88.9% compared to those under illuminated inorganic-N alone cultivation. Similarly, the mixture of arginine and urea was reported to have synergetic effect in promoting biomass and fucoxanthin production in the mixotrophic marine diatom *Phaeodactylum tricornutum* (Yang et al., 2022). Under the same total N concentration of 0.02 M, the mixture of tryptone and urea (1:1) or tryptone and sodium nitrate (1:1) were reported to be able to achieve higher *Phaeodactylum tricornutum* biomass productivity and fucoxanthin titer compared to single N source of tryptone (Wang et al., 2021b).

### Effects of light and type of N nutrition on cellular biochemical composition of *P. malhamensis*

**FIGURE 4:**
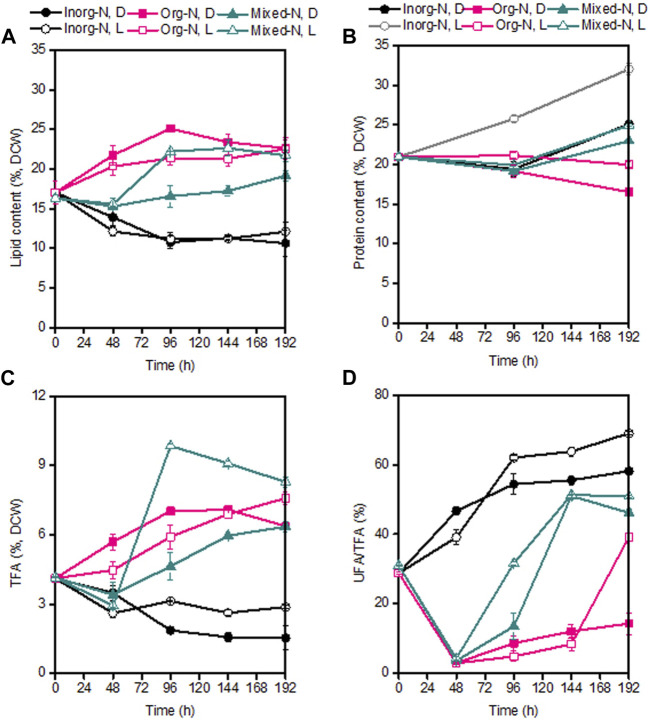
Comparison of lipid content **(A)**, protein content **(B)**, TFAs **(C)**, and ratio of UFA/TFA **(D)** under dark and illumination-coupled fermentation cultivation (white light and light intensity of 50 μmol m^−2^ s^−1^) with organic N (Org-N), inorganic N (Inorg-N) and mixed N nutrition. Data for **(A)** to **(D)** represent means ± SD (*n* = 3 biological replicates).

**FIGURE 5:**
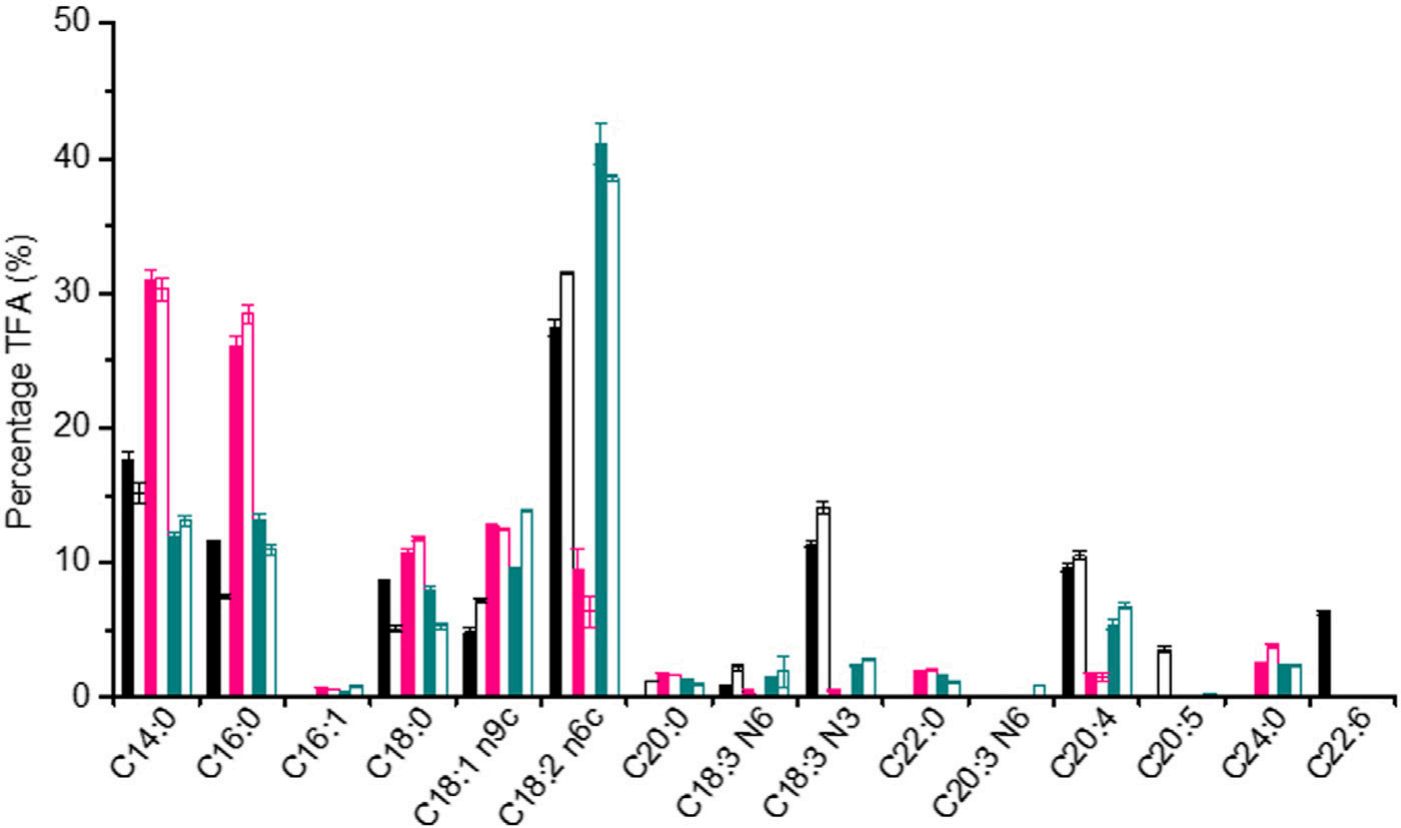
Comparison of fatty acid profiles of *P. malhamensis* samples under dark and illumination-coupled fermentation cultivation (white light and light intensity of 50 μmol m^−2^ s^−1^) with inorganic-N source (black column), organic-N (pink column), and mixed N (dark cyan column). Solid column: dark fermentation cultivation; blank column: illumination-coupled fermentation cultivation.

During the whole fermentation, the lipid content of *P. malhamensis* showed different changes under different type of N sources. Under inorganic-N alone, the lipid content dropped gradually. In contrast, lipid content under organic-N and mixed-N increased continuously. In addition, under the same medium N concentration, the maximum amount of lipid accumulation was observed under organic-N, while *P. malhamensis* grown under inorganic-N accumulated the minimum amount of lipid and more carbon flowed into the synthesis of β-1,3-glucan (Figures 1B, 4A). Similarly, under the same N concentration of 8.82 mM, the use of organic N yeast extract could result in much higher lipid production in *Tetraselmis* sp over inorganic nitrate-N (Kim et al., 2016). Under organic-N cultivation, the introduction of illumination induced the degradation of lipid and lower lipid content was observed compared to dark cultivation. In contrast, after 48 h’ mixed-N cultivation, the lipid contents under illumination were significantly higher than dark cultivation (*p* < 0.05). Under inorganic-N, light had little impact on total lipid accumulation, and the lipid content during the whole fermentation was very close between dark and illumination cultivation (Figure 4A). Under illuminated cultivation, higher total protein contents were observed for all types of N nutrition compared to dark cultivation (Figure 4B), which might be ascribed to the induction effect of light on promoting the synthesis of photosynthetic proteins (Fan et al., 2015). It was reported that when the *Chlorella* cells were transferred from heterotrophic cultivation to photoautotrophic cultivation, the cellular protein content increased markedly within the first 12 h, and the increment of protein was also associated with the concurrence of starch degradation (Fan et al., 2012). In contrast to the change of lipid content, total protein content of *P. malhamensis* under organic-N dropped gradually (Figures 4A,B). Under organic-N and mixed-N, total protein content reduced for the first 96 h and then increased continuously. The maximum amount accumulation of protein was obtained under illuminated inorganic-N cultivation, where the highest protein content at the end of fermentation reached 32% of DCW (Figure 4B). Total fatty acids (TFAs) content under different conditions showed similar changes to lipid content (Figures 4A,C). Moreover, relative to light, the type of N nutrition has much more significant influence on fatty acid profile, and larger ratios of unsaturated fatty acids (UFAs) (e.g., C18:2, C18:3, and C20:4) were observed under inorganic-N cultivation (Figure 5). Under illuminated inorganic-N nutrition cultivation, UFAs at the end of fermentation accounted for as high as 69% of TFAs, which was 76% higher than that under illuminated organic-N cultivation (Figure 4D). The influence of the type of N source on the fatty acids profile is species specific and has been demonstrated in several microalgae species (Lourenco et al., 2002). It was found that of all the tested algal species, higher percentages of total saturated fatty acids (SFAs) tended to occur equally in nitrate-N and ammonium-N media for *lsochrysis galbana*, *Phaeodactylum tricornutum*, *Nannochloropsis oculata* and *Synechococcus subsalsus*, while higher percentages of UFAs tended to be recorded in the urea-N media for *Hillea* sp., *Skeletonema costatum*, and *Tetraselmis gracilis* (Lourenco et al., 2002).

### Effects of different light spectra and light intensities on *P. malhamensis* growth, fucoxanthin and β-1,3-glucan production

**FIGURE 6:**
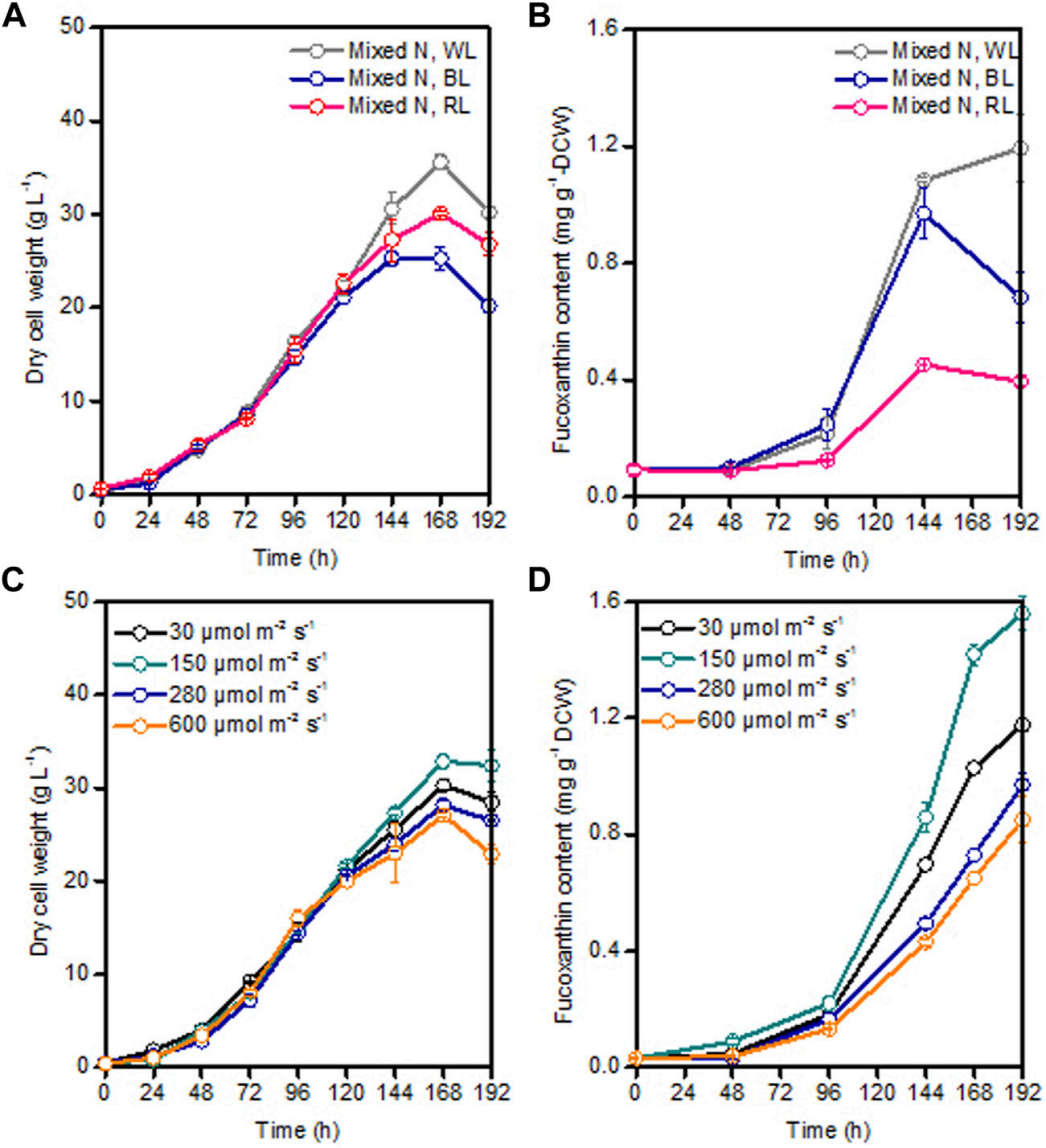
Effects of different light spectra with the same light intensity of 80 μmol m^−2^ s^−1^ and different light intensities (white light) on *P. malhamensis* growth **(A,C)**, and fucoxanthin content **(B,D)**. Data for **(A–D)** represent means ± SD (*n* = 3 biological replicates).

As the major light harvesting pigment, fucoxanthin mainly absorbs blue light and the mixture of blue and red light (Kuczynska et al., 2015). The effect of spectral quality on fucoxanthin production was reported to be species-specific. For example, blue light could achieve a higher fucoxanthin content compared to white and red light in the marine diatoms of *Thalassiosira weissflogii* and *Haslea ostrearia* (Mouget et al., 2004; Marella and Tiwari, 2020). However, highest fucoxanthin productivity in *Tisochrysis lutea* was found under the mixed red-blue-green light (i.e., closer to white light) (Gao et al., 2020). Under the same light intensity of 100 μmol m^−2^ s^−1^, *Cylindrotheca closterium* grown under blue light could produce comparable fucoxanthin productivity of 1.9 mg L^−1^ d^−1^ as white light (Wang et al., 2018). Differently, for the marine diatom *Odontella aurita*, red light was found to be more suitable for cell growth and fucoxanthin accumulation than blue light and white light (Zhang et al., 2022). Therefore, based on the previous study on light spectrum, three commonly used light spectra (i.e., white light, blue light, and red light) with the same average light intensity of 80 μmol m^−2^ s^−1^ were chosen to compare their influence on *P. malhamensis* growth and fucoxanthin production. Under these light spectra conditions, there was nearly no difference in *P. malhamensis* growth for the first 120 h (Figure 6A). The growth difference under different light spectra occurred mainly during the late growth phase (144–192 h), and higher biomass concentration was observed when illuminated with white light (Figure 6A). Under the same light intensity, white light was most suitable for the accumulation of fucoxanthin, and red light could only induce low level accumulation of fucoxanthin in *P. malhamensis* cells. The highest fucoxanthin content (0.54 mg g^−1^ DCW) under red light induction was less than half of that under white light (Figure 6B).

After obtaining the optimal light spectrum (white light) for fucoxanthin production, the impact of a wide light intensities from 30 to 600 μmol m^−2^ s^−1^ on *P. malhamensis* growth and fucoxanthin synthesis were investigated. It is widely accepted that low light intensity stimulates the accumulation of fucoxanthin while high light intensity favors to algal biomass accumulation (Gao et al., 2020; Mohamadnia et al., 2020; Li et al., 2022). For the first 120 h, the biomass concentrations of *P. malhamensis* under all different light intensities were very close (Figure 6C). However, the earliest decline of biomass concentration after 120 h was observed under high light intensity of 600 μmol m^−2^ s^−1^ and the maximum dry cell weight of 32.9 g L^−1^ was achieved under a moderate light intensity of 150 μmol m^−2^ s^−1^ (Figure 6C). Moreover, this light intensity (150 μmol m^−2^ s^−1^) was most suitable for fucoxanthin accumulation, and the highest fucoxanthin content under this light intensity could reach 1.56 mg g^−1^ (Figure 6D). When light intensity was increased from 150 to 600 μmol m^−2^ s^−1^, the accumulation of fucoxanthin decreased gradually. When illuminated at 600 μmol m^−2^ s^−1^, the highest fucoxanthin content was only 0.90 mg g^−1^, which was 57.7% of that obtained at 150 μmol m^−2^ s^−1^ (Figure 6D). Consequently, the maximum fucoxanthin yield and productivity under light intensity of 150 μmol m^−2^ s^−1^ reached 50.5 mg L^−1^ and 6.31 mg L^−1^ d^−1^, respectively.

Interestingly, the growth difference under different light spectra and light intensities occurred mainly during the late growth phase rather than the initial growth phase (Figure 6). The possible reason might be as follows. Under dark heterotrophic cultivation, as the *P. malhamensis* cells use glucose as their carbon source and energy source to support cell growth, they do not require to establish their photosynthetic system, which could be reflected from the extremely low fucoxanthin content during the whole dark cultivation as well as the observed strong positively linear correlation between fucoxanthin and chlorophyll a content (Figure 1C, 2). When inoculating the *P. malhamensis* cells from dark flask cultivation to illuminated fermentation cultivation, the observed fucoxanthin content during the initial growth phase before 96 h was also very low, indicating that the photosynthetic system in *P. malhamensis* cells was not established during this phase. It appears that the *P. malhamensis* cells during this initial growth phase might not be sensitive to light due to their unestablished photosynthetic system. So even though they were cultured in the presence of light, they exhibited nearly the same growth with the cells under dark cultivation during this initial growth phase (Figure 1A). Also for this reason, very close cell growth during the initial growth phase was observed under different illumination conditions including different light spectra and light intensities (Figures 6A,C). However, once the photosynthetic system in *P. malhamensis* cells was well established, their growth might be affected by the change of light conditions. It has been found that the rapid increase of fucoxanthin content occurred mainly during the late growth phase after 96 h (Figure 6), indicating that the photosynthetic system of *P. malhamensis* cells has been well established during this late growth phase. Therefore, the growth difference under different illumination conditions was observed mainly during the late growth phase.

### Comparison of fucoxanthin production by *P. malhamensis* with other fucoxanthin-rich microalgae

Table 1 compared the highest fucoxanthin productivity, fucoxanthin content and biomass concentration of *P. malhamensis* with other well-studied fucoxanthin-rich microalgae. As shown in Table 1, photoautotrophic culture was the main culture mode for most of the fucoxanthin-rich microalgae species. Commercial-scale fucoxanthin production in photobioreactors was usually limited by the difficulty to achieve high biomass concentration (Lu et al., 2018). For most fucoxanthin-rich microalgae, their highest biomass concentrations in laboratory culture were generally around 1–3 g L^−1^ (Table 1). Even though some microalgae (e.g., *Odontella aurita*) in laboratory culture could reached a relatively higher biomass concentration of 5.65 g L^−1^ (Table 1), great decline in fucoxanthin content and biomass concentration was observed after a 12-fold scale-up cultivation (Zhang et al., 2022). So far, the reported highest fucoxanthin productivity under photoautotrophic culture was less than 10 mg L^−1^ d^−1^ (Table 1).

**TABLE 1:** Overview of the maximum biomass concentration, fucoxanthin content and productivity of various fucoxanthin-rich microalgae.

Microalgae species	Culture modes	Maximum biomass conc. (g L^−1^)	Maximum fucoxanthin content (mg g^−1^)	Maximum fucoxanthin productivity (mg L^−1^ d^−1^)	References
*Tisochrysis lutea*	Photoautotrophic culture	1.23	10.01	9.81	Gao et al. (2020)
*Pavlova lutheri*	Photoautotrophic culture	2.20	20.86	4.88	Kanamoto et al. (2021)
*Isochrysis zhangjiangensis*	Photoautotrophic culture	2.00	22.60	3.06	Li et al. (2022)
*Phaeodactylum tricornutum*	Photoautotrophic culture	0.37	59.20	2.30	McClure et al. (2018)
*Thalassiosira weissflogii*	Photoautotrophic culture	3.20	9.00	5.10	Marella & Tiwari, (2020)
*Odontella aurita*	Photoautotrophic culture	5.65	16.2	9.41	Zhang et al. (2022)
*Sellaphora minima*	Photoautotrophic culture	1.71	7.60	1.20	Gerin et al. (2020)
*Nitzschia palea*	Photoautotrophic culture	1.19	5.70	0.60	Gerin et al. (2020)
*Chaetoceros gracilis*	Photoautotrophic culture	0.80	15.40	3.82	Tachihana et al. (2020)
*Mallomonas sp*	Photoautotrophic culture	3.75	26.60	7.13	Petrushkina et al. (2017)
*Phaeodactylum tricornutum*	Mixotrophic culture	3.73	13.00	8.22	Yang & Wei, (2020)
*Nitzschia laevis*	Hetero-Photoautotrophic culture	17.25	12.00	16.50	Lu et al. (2018)
*Poterioochromonas malhamensi*	Illumination-coupled fermentation culture	32.9	1.56	6.31	This study

Heterotrophic culture offers the possibility to achieve high biomass concentration and stable scale-up cultivation of microalgae in well-controlled fermentor is readily achieved. Under 1,000-L pilot scale-up cultivation, the obtained maximum biomass concentrations of both *Scenedesmus acuminatus* and *Chlorella sorokiniana* were very close to those achieved in 7.5-L bench-scale fermentor cultivation (Jin et al., 2020; Jin et al., 2021). However, to date, only limited studies have been reported on fucoxanthin production through heterotrophic cultivation. Recently, the marine diatom *Nitzschia laevis* was found to be able to growth heterotrophically and accumulate fucoxanthin without the presence of light. By using a two-stage hetero-photoautotrophic cultivation strategy with fermentors and bubbling column bioreactors, a high biomass concentration of 17.25 g L^−1^ and thereby a superior fucoxanthin productivity of 16.5 mg L^−1^ d^−1^ was achieved by *N. laevis* (Table 1). Obviously, this record fucoxanthin productivity relies heavily on the tremendous improvement of biomass concentration obtained through heterotrophic cultivation. Therefore, the screening and exploitation of new fucoxanthin-rich microalgae resources capable of heterotrophic growth will be very important for markedly improvement of current fucoxanthin productivity.

In this study, we achieved the highest *P. malhamensis* biomass concentration of 32.9 g L^−1^, which is 90% higher than that of *N. laevis*. Despite a much lower maximum fucoxanthin content, the achieved maximum fucoxanthin productivity (6.31 mg L^−1^ d^−1^) by *P. malhamensis* was still comparable to and even higher than the reported highest levels by most fucoxanthin-rich microalgae *via* photoautotrophic cultivation (Table 1). Obviously, the illumination-coupled fermentation cultivation mode combined the advantages of both heterotrophic fermentation on achieving high biomass concentration and light on inducing fucoxanthin accumulation. Recently, pilot-scale photo-fermentor with inner illumination by equipped with LEDs has been designed and applied in *Chlorella pyrenoidosa* to recover nitrate from synthetic wastewater (Wang et al., 2022). Like industrial fermentor, important fermentation process parameters (e.g., pH, temperature, and DO) in closed photo-fermentation system could be well controlled and contamination risk could also be greatly reduced. Therefore, once a much higher fucoxanthin content and productivity was achieved in laboratory scale culture system, it is promising that cultivating *P. malhamensis* in large-scale photo-fermentor for commercial fucoxanthin production. It also should be pointed out that the introduction of artificial light in conventional fermentor will increase the total cost of equipment investment. Moreover, extra heat release produced by illumination will increase the burden of cooling and also increase the total power cost. However, it is still economically feasible when photo-fermentation system is used for high-value fucoxanthin production.

## Conclusion

A novel fucoxanthin production mode by coupling illumination with heterotrophic fermentation process was proposed in the present study. Under illuminated high-cell-density cultivation, the application of combined organic and inorganic N nutrition could improve *P. malhamensis* growth while maintaining a high fucoxanthin accumulation. White light was found to be the optimal light quality and a moderate light intensity of 150 μmol m^−2^ s^−1^ was most suitable for fucoxanthin production. Finally, the maximum fucoxanthin yield of 50.5 mg L^−1^ and productivity of 6.31 mg L^−1^ d^−1^ were achieved by coupling heterotrophic high-cell-density fermentation with extra illumination.

## Data Availability

The original contributions presented in the study are included in the article/Supplementary Material, further inquiries can be directed to the corresponding author.

## Supplementary material

SUPPLEMENTARY FIGURE S1 Experimental photographs of dark and illumination-coupled **(B)** fermentation cultivation.

SUPPLEMENTARY FIGURE S2 Emission light spectrum of white light , red light **(B)** and blue light **(C)**.

SUPPLEMENTARY FIGURE S3 HPLC chromatogram of pigment samples from dark 48 h cultivation, dark 120 h cultivation, light 120 h cultivation, and standard pigment sample.

SUPPLEMENTARY FIGURE S4 Comparison of culture samples from dark and light cultivation with inorganic and organic N sources.
