# Supplementary material for: Effective fucoxanthin production in the flagellate alga Poterioochromonas malhamensis by coupling heterotrophic high-cell-density fermentation with illumination
Source: Front Bioeng Biotechnol. 2022 Dec 2;10:1074850. doi: 10.3389/fbioe.2022.1074850 (PMC9755745; doi:10.3389/fbioe.2022.1074850)
Supplement: Supplementary file 1 [file DataSheet1.docx]

**Effective fucoxanthin production in the flagellate alga *Poterioochromonas malhamensis* by coupling heterotrophic high-cell-density fermentation with illumination**

Hu Jin*^†1^, Yufen Guo^†2^, Yanhua Li^†1^, Baofeng Chen^1^, Haiyan Ma^1^, Hongxia Wang^1^, Lan Wang^1^, Danni Yuan^3^

^1^Institute of Hydrobiology, Chinese Academy of Sciences, Wuhan 430072, China

^2^School of Life Science and Food Engineering, Huaiyin Institute of Technology, Huaian 223003, China

^3^School of Environmental Ecology and Biological Engineering, Wuhan Institute of Technology, Wuhan 430205, China

^†^ Contributed equally to this work

* Corresponding to Hu Jin ([jinhu@ihb.ac.cn](mailto:jinhu@ihb.ac.cn))

**Figure 1.** Experimental photographs of dark (A) and illumination-coupled (B) fermentation cultivation

**Figure 2.** Emission light spectra of white light (A), red light (B) and blue light (C)

**Figure 3.** HPLC chromatogram of pigment samples from dark 48 h cultivation, dark 120 h cultivation, light 120 h cultivation, and standard pigment sample

**Figure 4.** Comparison of culture samples from dark and light cultivation with inorganic and organic N sources
